# The relationship between self-assessment living standard and mental health among the older in rural China: the mediating role of sleep quality

**DOI:** 10.1186/s12889-023-15157-1

**Published:** 2023-03-08

**Authors:** Beibei Zhang, Xianwen Wang, Song Liu, Min Zhang, Xinran He, Weizheng Zhu, Hong Ding

**Affiliations:** 1 Department of Health Service Management, School of Health Management, Anhui Medical University, Hefei, Anhui China; 2 School of Public Health and Health Management, Anhui Medical College, No. 81 Meishan Road, Hefei, 230032 Anhui China

**Keywords:** Self-assessment, Living standard, Mental health, Sleep quality, Older

## Abstract

**Background and objective:**

Mental health imbalance are the main cause of anxiety, depression and happiness reduction in the older. Self-assessment living standard and sleep quality are both influencing factors of mental health. Meantime, self-assessment living standard has an impact on sleep quality. But there’s no research on the relationship between the three, we conducted this study to explore the relationship between self-assessment living standard and mental health and the mediating role of sleep quality among the older in rural areas of China.

**Methods:**

Using typical field sampling method, M County, Anhui Province was selected as the investigation site, and a total of 1223 respondents were selected. With the help of questionnaires enclosing respondents’ sociodemographics information, 12 Items General Health Questionnaire (GHQ-12) and Pittsburgh Sleep Quality Scale (PSQI), face-to-face interviews were used to collect data. Bootstrap test was used for data analysis.

**Results:**

The results showed that the age of the respondents ranged from 60 to 99 years, with an average age of (66.53 ± 6.77) years, the proportion of the older with a tendency to mental health problems was 24.7%. Most of the older people’s self-assessment living standard was normal (average score was 2.89 ± 0.726), accounting for 59.3% of the total. The average sleep quality score was (6.97 ± 4.066), and 2.5% of the respondents reported serious sleep problems. older with low self- assessment living standards were more likely to report a higher propensity for psychological problems (*β* = 0.420, *P* < 0.001) and poorer sleep quality (*β* = 0.608, *P* < 0.001) than older with high self- assessment living standards. Mental health of the older may be related to sleep quality (*β* = 0.117, *P* < 0.001). In addition, the effect of self- assessment living standard on mental health was significantly mediated by sleep quality (*β* = 0.071, *P* < 0.001).

**Conclusion:**

Mental health is associated with self-assessment living standard, with this association mediated by sleep quality. A reasonable mechanism needs to be established to improve self-assessment living standard and sleep quality.

## Introduction

According to the results of China’s seventh census, the number of Chinese aged 60 and above has reached 264 million, accounting for 18.70% of the total population. The population aged 65 and above is about 190 million, accounting for 13.50% [1]. With the deepening of the aging degree in China, the psychological problems of the older have become increasingly prominent due to the changes in social and family structure, physical aging, the influence of diseases, and inadequate support system [2]. The data showed that the proportion of the older with mental problems in various regions of China ranged from 2.26 to 69.68% [3], Among them, the proportion of the older in rural areas with mental health problems is 1.76 times that of the older in urban areas [4], meanwhile, the life security, physical function and social support of the older in rural areas are significantly lower than those in urban areas [5]. According to the China Statistical Yearbook, the per capita disposable income of rural residents was 18,931 yuan in 2021, while that of urban residents was 42,412 yuan. Urban residents’ per capita disposable income was 2.5 times that of rural residents [6]. Although the government provides certain security policies for the elderly, the national standard of 55 yuan per person per month for elderly people over 60 without employee pension insurance in China. Most rural residents do not enjoy the fair treatment of social endowment insurance. There are differences in pension funds paid and received by rural and urban residents. The pension funds paid by rural residents account for a larger proportion of their income, but the insurance funds received by rural residents are lower than those of urban residents [7].

WHO defines health as a state of complete physical, mental and social adaptation, not just no disease or infirmity [8]. Psychological imbalance not only has a direct impact on physical health, but also has an indirect impact on social support and quality of life, bringing a huge burden of disease [9]. Studies have shown that age, economic status, physical status, social support, social role and lifestyle change are the main factors affecting the mental health of the older [10]. Research shows that rural residents in health spending suffered greater economic burden, its proportion is 2.4 times that of urban residents [11], at the same time, another study found, catastrophic health expenditure of Chinese rural areas was obviously higher than that of urban areas [12], studies have shown that China’s rural older people’s quality of life in rural areas and the low state of the economy [13]. Jiang Haochen’s research proved that compared with the older with a more affluent living standard, the older with a poorer living standard reported worse mental health [1], and reveal the self- assessment living standards may have an important impact on the mental health of the older population. Research pointed out that in addition to the objective measurement of economic level, people’s subjective economic pressure measurement on the personal happiness and satisfaction occupy more important position [14], of the living standards of their subjective evaluation mainly from compare yourself with other living conditions, especially in rural areas of China the older by education degree is generally low, less mental recreation makes it easier to compare oneself with others. If one continues to believe that his or her standard of living is below the average or reference level, this long-term gap will indirectly affect his or her mental health. At present, China has achieved comprehensive poverty alleviation, and absolute poverty, which is measured by meeting basic survival needs, has been eliminated. However, the identification of relative poverty, which reflects the gap between individual economy, living conditions and local average living standards, has received little attention [15]. In addition, relevant studies on the living standards of the older in rural China focus more on objective evaluation of living standards, and less on subjective evaluation [16–18].

Self-assessment living standard is a subjective evaluation of their own living standard, which reflects the satisfaction and expectation of the older to their living conditions. Evidence shows that the worse self-assessment living standard contributes to poor sleep health. For example, due to the rapid growth of social economy, rising price level, a large number of young and middle-aged people go out to work, the rural older people often feel helpless and declined the standard of living, think about things at night, and then suffer from poor sleep quality [19]. In turn, poor sleep quality will affect the older’s daily activities, social interactions and attitudes toward life, resulting in lower life satisfaction and worse self-assessment living standards [19].

Sleep quality usually declines gradually with age [20], and some older people may suffer from sleep disorders. The incidence of sleep disorders among the older over 60 years old in China is 30% ~ 40% [21]. Sleep disorders are mainly manifested as difficulty in falling asleep and maintaining sleep, which leads to sleep deficiency and fatigue, and patients find it difficult to recover from sleep [22]. A large number of studies have shown that poor sleep quality will not only increase the occurrence of chronic diseases, but also increase the risk of death [23–25]. According to the view of chronobiology theory, the biorhythmic system is closely related to many diseases, and regular sleep contributes to the stability of human psychological functions [26]. If the body’s sleep time changes and the body’s functions are disordered, it will affect the disorder of emotional function and lead to a decline in psychological conditions. Studies have shown that there is a significant correlation between sleep quality and mental health, and sleep disorders (such as insomnia, narcolepsy, sleep apnea and circadian complaints) have a high comorbidity rate with depression and anxiety [27, 28], optimizing sleep quality can promote mental health [29]. Meanwhile, the older with poor sleep quality are more likely to suffer from hypertension, depression and other diseases [25]. At present, there are many studies on the influencing factors of sleep disorders in the older in China. For example, moderate exercise is beneficial to improve the sleep quality of the older [30]. Interpersonal relationship can affect the sleep quality of the older by affecting their mood [31]. However, the potential causes of sleep quality among the older in China have not been fully appreciated.

Given that sleep quality is one of the important predictors of mental health [32]. Improving self-assessment living standards in older may reduce mental illness by improving sleep quality. However, little is known about the mechanisms that link self-assessment living standard and mental health prospectively. There are no studies that have tested whether poor sleep quality mediates the relation between self-assessment living standard and mental health [33–35]. In the context of the rapid development of aging society has become the basic national conditions of China, the mediation of the impact of self-assessment of living standards on mental health has become an issue that needs to be studied in the prevention of psychological abnormalities in the older. Therefore, we conducted a cross-sectional study to uncover the relationship between self-assessment living standards and mental health among older people in rural China, and to consider the role of sleep quality in this study. This study can provide a theoretical basis for improving the sleep quality and mental health of rural elderly.

## Methods

### Study design and data collection

From July to September 2021, we conducted a cross-sectional survey in M County, Anhui Province, central China. M county is a pilot county of compact county medical community. The local county and village medical and health service system is sound and relevant departments have strong coordination, providing good external conditions for the research work.

Two towns in M County, Anhui Province, China were randomly selected, and 5 villages were randomly selected in each town. M County is a typical rural area in central China. Its level of economic development and per capita income are below the average level of China. In 2021, the per capita disposable income of permanent residents in M County was 24,344 yuan, and the per capita disposable income of permanent rural residents was 17,221 yuan [36]. The annual per capita disposable income of Chinese residents was 35,128 yuan [37], the economic level of the older in M County was significantly lower than the national average.

The older ≥ 60 years old in the villages were investigated. The selection criteria of the research objects were as follows: (1) subjects aged 60 years and above (according to Article 2 of the Law on the Protection of the Rights and Interests of the older, the age of the older is 60 years old); (2) Subjects who have lived there for at least 1 year at the time of investigation. Exclusion criteria included sensory or cognitive impairment, contraindications to physical activity, a medical diagnosis of a primary sleep disorder (for example, sleep apnea or primary insomnia). Before the investigation, all subjects were told the purpose and procedure of the study orally. The investigators were all postgraduates from Anhui Medical University who had received unified training and doctors from local township health centers. Each subject was visited and interviewed face-to-face. A total of 1223 older people were surveyed, of whom 1188 completed the survey, with an effective response rate of 97.14% (1188/1223).

### Measurement of self-assessment living standard

This study used self-assessment living standard to measure the living standard of the older. The respondents were asked “What is your living standard in the local area?“, the answers were divided into five levels: “very good”, “good”, “average”, “poor” and “very poor”, with a value of 1, 2, 3, 4 and 5 respectively. The higher the score was, the lower the self-assessment living standard.

### Measurement of sleep quality

The Pittsburgh Sleep Quality Scale (PSQI) was used in this study. PSQI was developed by Buysse et al. [38] for self-assessment of sleep in the past 1 month. The scale consists of 7 dimensions, including subjective sleep quality, sleep time, sleep time, sleep efficiency, sleep disorders, sleep drugs, and daytime dysfunction. Each dimension is 0 ~ 3 points, and the cumulative score is the total score. The lower the score, the better the sleep, the cumulative score of 7 or more indicating sleep disturbance [39]. The Cronbachα coefficient of the scale was 0.77, the half-fold reliability was 0.83, and the structural validity was 0.63–0.91, indicating that the scale had good reliability and validity and was widely used [38, 40].

### Measurement of mental health

Mental health was measured using the 12 Items General Health Questionnaire (GHQ-12), a self-assessment screening tool that has been successfully applied to the Chinese sample. There are 12 items in the questionnaire, and the answers to each item are divided into four options. The first two items are counted as 0 points, and the last two items are counted as 1 point. The total score ranges from 0 to 12 points. The higher the GHQ-12 score, the higher the risk of developing psychological disorders [41]. The Cronbach’s alpha coefficient of GHQ-12 was 0.793.

### Statistical analysis

First, we used the Chi-square test to examine differences in mental health among older adults with different living standards and quality of sleep. Rates and percentages are used to describe the demographic characteristics of different groups of subjects.

**Fig. 1:**
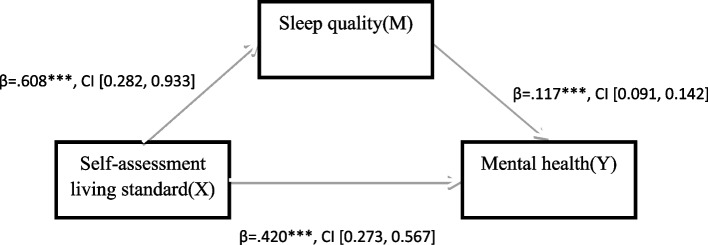
Mediating model of self-assessment living standard, sleep quality and mental health

Next, Pearson correlation analysis was used to test the correlation between variables. In order to further explore the specific role path of sleep quality in the mediating effect of self-assessment living standard on mental health, this study adopted the mediating effect test method proposed by Hayes [42], taking self-assessment living standard as independent variable, mental health as dependent variable and sleep quality as intermediary variable to test the significance of the mediating effect. Mediation test Model 4, developed by Hayes based on the SPSS macro program PROCESS, uses the non-parametric percentage Bootstrap method with bias correction to extract an estimated 95% confidence interval repeatedly for 5000 times. When the confidence interval of each path coefficient does not include 0, it indicates that the mediation effect is significant. According to the test results, the mediation effect path analysis model is drawn, as shown in Fig. 1.

## Results

### Characteristics of participants

Table 1 describes the general demographic characteristics of the respondents. The study involved 1,188 participants, all participants are between the ages of 60–99 (mean age = 66.53 years, SD = 6.577). The average of self-assessment living standard is (2.89 ± 0.726), and the sleep quality was (6.97 ± 4.066), mental health is (2.08 ± 1.90). Of these participants, 895 reported good mental health and 293 reported poor mental health. There are statistically significant differences between the two groups in basic demographic characteristics such as gender, education level, living status, working status, chronic diseases and hospitalization, and sleep quality. Among the 895 subjects with good mental health, 50.72% (454/895) are male, 36.65% (328/895) are aged between 60 and 69, 21.90% (196/895) lived with their spouse, and 85.70% (767/895) had not seen a doctor in the last two weeks. 60.56% (542/895) had not been hospitalized in the past one year.

**Table 1:** General characteristics of the respondents and Chi-square test results of influencing factors of mental health in rural older people (*N* = 1188)

	Total (*N* = 1188)	Mental health		χ^2^	*p-value*
		Good (*N* = 895)	Poor (*N* = 293)		
**Gender**				5.269	0.022
Male	580(49.3)	454(50.73)	126(43.00)		
Female	608(50.7)	441(49.27)	167(57.00)		
**Age(years)**				0.027	0.986
60–69	435(36.6)	328(36.65)	107(36.52)		
70–79	558(47.0)	421(47.04)	137(46.76)		
≥ 80	195(16.4)	146(16.31)	49(16.72)		
**Married status**				1.613	0.656
Married	841(70.8)	642(71.73)	199(67.92)		
Divorced	8(0.7)	6 (0.67)	2(0.68)		
Widowed	289(24.3)	21(2.3)	79(26.96)		
Others	50(4.2)	37(4.13)	13(4.44)		
**Education level**				11.561	< 0.001
Primary and below	974(81.7)	712(79.55)	259(88.40)		
Junior and above	217(18.3)	183(20.45)	34(11.60)		
**Employment status**				34.228	< 0.001
Normal work	265(22.3)	197(22.01)	68(23.21)		
Half work	380(32.0)	309(34.53)	71(24.23)		
Housework	316(26.6)	250(27.93)	66(22.53)		
Don’t work	224(18.9)	137(15.31)	87(29.69)		
Other	3(0.3)	2(0.22)	1(0.34)		
**Living style**				7.868	0.020
Living alone	250(21.0)	485(54.19)	145(49.49)		
Living with spouse	630(53.0)	196(21.90)	54(18.43)		
Other	308(25.9)	214(23.91)	94(32.08)		
**Living standard**				27.189	< 0.001
Good	282(23.7)	239(26.70)	44(15.02)		
Common	705(59.3)	529(59.11)	176(60.07)		
Bad	308(25.9)	128(14.30)	73(24.91)		
**Chronic diseases**				7.688	0.006
Yes	915(77.0)	672(75.08)	243(82.94)		
No	273(23.0)	223(24.92)	50(17.06)		
**Physical discomfort**				9.265	0.002
**(Within two weeks)**					
Yes	996(83.8)	767(85.70)	229(78.16)		
No	192(16.2)	128(14.30)	6(2.05)		
** Hospitalization**				12.490	< 0.001
**(Within a year)**					
Yes	685(57.7)	524(58.55)	143(48.81)		
No	503(42.3)	353(39.44)	150(51.19)		
**Sleep quality**				29.578	< 0.001
Good	637(53.61)	579(64.69)	58(19.80)		
Bad	551(46.38)	316(35.30)	235(80.2)		

### The relationship between living standard, sleep quality and mental health

Pearson correlation analysis is conducted on the data of self- assessment living standard, sleep quality and mental health scales, and the results are shown in Table 2. The score of mental health status is significantly positively correlated with the score of self- assessment living standard and sleep quality.

In Table 3, bootstrap test analysis results showed that self-assessment living standard had a significant direct impact on mental health (β = 0.420, 95%CI 0.273–0.567). older people with higher self-assessment living standards are likely to report higher levels of mental health. Meanwhile, self-assessment living standard is significantly associated with sleep quality: higher self-assessment living standard is associated with better sleep quality compared with lower self-assessment living standard (β = 0.608, 95%CI 0.282–0.933). There is also a link between sleep quality and mental health. Higher sleep quality scores are associated with higher mental health level (β = 0.117, 95%CI 0.091–0.142). Based on the results, a path map of self-assessment living standards, sleep quality and mental health is drawn, as shown in Fig. 1.

**Table 2:** Correlation analysis of subjective evaluation of living standard, sleep quality and mental health

	Self-assessment of living standards	Sleep quality	Mental health
Self-assessment of living standards	1		
Sleep quality	0.107***	1	
Mental health	0.178***	0.265***	1

for: ***means *P*＜0.001

**Table 3:** Bootstrap test of self- assessment living standard, sleep quality and mental health

Dependent variable	Predictive variable	Standardized regression coefficient	SE	T	95%CI		R²	F	P
					LLCI	ULCI			
Sleep quality	Living standard	0.608	0.166	3.662	0.282	0.933	0.012	13.411	0.000
Mental health	Sleep quality	0.117	0.013	8.807	0.091	0.142	0.095	60.463	0.000
	Living standard	0.420	0.077	6.377	0.273	0.567	0.340	40.67	0.000

### Mediating effect analysis of self-assessment living standard, sleep quality and mental health

Sleep quality is a potential mediator in the association between self-assessment living standards and mental health (β = 0.071, 95%CI 0.021–0.099). Bootstrap test results showed that the 95%CI of direct and indirect effects of self-assessment living standard on mental health score did not include 0. The results indicate that sleep quality plays a partial mediating role in the relationship between the self-assessment living standard and mental health of the older in rural areas, and the partial mediating effect value is 0.071, accounting for 14.46% of the total effect. The specific results are shown in Table 4.

**Table 4:** The mediating effect of sleep quality on self-assessment living standard and mental health

	Effect	Boot SE	*t*	*P*	95%CI	
The total effect X->M->Y	0.491	0.077	6.377	0.000	0.339	0.642
Direct effect X->Y	0.420	0.075	5.604	0.000	0.273	0.567
Indirect effect M->Y	0.071	0.023			0.029	0.119

## Discussion

This study proved the relationship among self-assessment living standard, mental health and sleep quality among the older in rural areas of Anhui Province. The results showed that self-assessment living standard was closely related to mental health, and the older with low self-assessment living standard had a higher risk of psychological problems. However, this correlation occurs through both direct and indirect effects. Sleep quality played a significant partially mediating role between self-assessment living standard and mental health.

### Rural older self-assessment living standard, sleep quality and mental health status

Among the 1,188 respondents, 702(59.34%) thought their living standard was average, 202(16.92%) thought their living standard was poor or even very poor, and 284(23.74%) thought their living standard was good, among which the proportion of self-assessment was average or poor was significantly higher than the research results of Jiang Haochen [43]. The reason may be that the regional distribution of the survey objects and the total number of samples are different. Meanwhile, the economic status and medical level of rural areas are lower than the national level [43]. Some studies have classified the lifestyle of the older in China into four types: survival type, healthy type, risk type and mixed type, with 45%, 25%, 13% and 17% respectively. The life style of the older in rural Areas of China is mainly subsistence lifestyle [44]. They control the living cost and have few social participation behaviors, mostly watching TV and listening to radio, and less intake of fresh fruits and fish in daily life, which may be an important reason for their low self-assessment of living standard [45].

The results of this study show that 24.7% of the rural older have a tendency to have mental health problems, which is similar to 18.5%~24.47% of the general older population [46–48]. Compared with the urban older, the rural older in China have less financial resources, social support, family companionship, etc., and relatively overlapping living environment, which may have a negative impact on their mental health [49]. Gender, education level and working state have statistical significance to mental health difference, which is consistent with the research conclusions of Liang Xiaoli ; Zhang Pei [50, 51].

Compared with men, women are more sensitive to emotions and more prone to mental problems [52]. The older with high education level have higher cognitive ability and health awareness, and can enrich themselves by reading books, reading newspapers and participating in social activities, so as to better cope with difficulties. On the other hand, the older with a low education level, limited by their cognitive level, have a poor ability to judge things and accept new things, and are prone to suffer from inferiority complex, loneliness and other psychological problems. Their enjoyment of life is relatively limited, which is more likely to cause psychological problems [53]. Older who are able to work regularly tend to report better mental health, possibly because working in rural areas is the norm. Older people who are unable to work normally always experience feelings of guilt, particularly if they are not able to work at all, they will see themselves as a burden on their families [54].

In addition, 48.4% of participants with PSQI scores above 7, and 2.5% of participants reported serious sleep problems, which was higher than the results of Ding Kunxiang’s study [55]. This may be due to the different time points we surveyed and changes in the social environment in rural areas [56].

### The effect of self-assessment living standard on mental health

Previous studies have observed a correlation between self-assessment living standards and mental health [57–59]. Previous studies have revealed the impact of poverty on the mental health of the older. For example, one research (2012) found that poverty was significantly correlated with cognitive impairment and depression in the older in India [60]. A study (2017) on the older in rural China shows that the mental health status of the older in poor families is worse [61]. A scholar. (2021) conducted a study on the older aged 65 and above in China, which verified that the older with lower self-assessment living standards had more severe negative psychological emotions [62]. The negative impact of lower living standards on mental health may come from the negative impact of less economic foundation and resources on individuals’ physical health and social behavior, or the poverty-related living environment may lead to more stress and negative emotions, thus affecting mental health [1]. In addition, social comparison theory believes that social comparison is intra-group and inter-group comparison, and the latter has a more obvious impact on individual psychological development [63]. However, these studies use different participant groups (e.g., young adults) or analytical methods (e.g., traditional regression and correlation). Although those studies differed from ours in terms of specific details, the results regarding the negative relationship between self-assessment living standard and mental health were consistent, which confirms the results of our study.

### The impact of self-assessment living standards on sleep quality

This study found that self-assessment living standard was associated with an increased likelihood of high sleep quality among the older in rural areas in Anhui province. A study conducted in Yunnan Province, China, showed that older people in rural areas with lower family property have a higher likelihood of sleep disorders [64]. It may be related to their sensitive emotions. The older with lower living standards are more likely to have negative thoughts and to have random thoughts before going to sleep, which affects their sleep. Low level of self-reported life means not only a single economic sources, less material resources, and poor living environment, also means that more stressful life events and negative mood [65], which will result in its sleep problems obviously increased, low level of self-reported life will bring such as difficulty falling asleep, wake up, wake up at night and having nightmares and other sleep problems.

### The mediating role of sleep quality in self-assessment living standard and mental health

This study found that sleep quality was the mediating variable between the self-assessment living standard and mental health of the rural older, playing a partial mediating role, accounting for 14.46% of the total effect. Specifically, older who reported low self-assessment living standards are more likely to suffer from poor sleep quality, which in turn led to worse mental health over time. According to the theory of chronobiology [66], the onset of mental diseases is closely related to the biorhythmic system, and the elderly who report their poor living standards are prone to cranky thoughts at night, resulting in the disorder of the sleep system, destroying the normal regulatory mechanism of the human body, and increasing the risk of psychological problems.Earlier studies have also confirmed this conclusion. In a study of German communities and students found that when individuals’ sleep quality and mental health are not healthy, measures to improve sleep can better promote the improvement of mental health [63]. Another study found that poor sleep quality is associated with increased incidence of violations, aggression, depression and anxiety [67]. One study in China [68] shows that when the proportion of children going out is high, the negative missing time effect is dominant, which is not conducive to the improvement of parents’ health. Possible explanations for this result is that although China has comprehensive poverty alleviation, rural residents general living standards improve gradually, but the income of the rural older people in China still is generally low, cultural life still relatively monotonous and boring [69], coupled with the decline in physiological function, relative lack of medical resources, children migrant workers and other factors, It will have a negative impact on their economic status and living standards for a long time. At the same time, they are easy to fall into sleep difficulties, easy to wake up, nightmares and other sleep disorders, leading to their inability to relieve mental stress through sleep, resulting in psychological problems. When the quality of sleep is poor in the older, it will also affect their self-rated living standards [70]. Poor sleep quality will affect the older’s daily activities, social interactions and attitudes toward life, resulting in lower life satisfaction and worse self-assessment living standards [71]. Therefore, China can help prevent sleep and psychological problems in the older by strengthening the training of Primary healthcare workers in this therapy.

### Advantages and limitations

Advantages: First, the effective response rate of this study is 99.00% (1188/1223), as we all know, studies with higher effective response rates were more reliable. Secondly, we used internationally recognized measurement questionnaires to make objective measurements of the study subjects. In addition, this is the first study to examine the relationship between the three variables and the mediating role of sleep quality in the older population in Anhui Province.

However, this study also has the following limitations: First, self-assessment living standards, sleep quality, and mental health were measured through questionnaires, which means that self-reported biases may affect the results. At the same time, because the measurement of the self-assessment living standard of the older is single, the reliability of the answer will be reduced, which may impact the research results. Second, since this study is a cross-sectional study, although there is a correlation between self-assessment living standards, sleep quality and mental health, it is difficult to determine the causal association. Finally, the investigation objects of this study only cover rural areas of Anhui Province, and the extensibility of the results of this study is limited by factors such as economic development and cultural background.

## Conclusion

Our research shows that self-assessment low living standards and poor sleep quality can exacerbate psychological problems. In addition, sleep quality mediates the relationship between self-assessment living standards and mental health. Our results may help alleviate psychological problems and improve sleep quality of rural older, and provide information for clinical prevention of diseases. It is suggested that the government and society pay more attention to the health of the rural older, improve the rural older security system, and improve the level of security.

## Data Availability

The datasets generated during the study are not publicly available due to an ethical restriction but are available from the corresponding author on reasonable request.
